# Systematic review: a systems innovation perspective on barriers and facilitators for the implementation of healthy food-store interventions

**DOI:** 10.1186/s12966-019-0867-5

**Published:** 2019-11-21

**Authors:** Cédric N. H. Middel, Tjerk Jan Schuitmaker-Warnaar, Joreintje D. Mackenbach, Jacqueline E. W. Broerse

**Affiliations:** 1 Athena Institute, Faculty of Science, VU University, De Boelelaan 1085, 1081 HV Amsterdam, Netherlands; 2 Department of Epidemiology and Biostatistics, Amsterdam University Medical Centres, VU University Medical Center, Amsterdam, the Netherlands

**Keywords:** Cardiovascular diseases, Diabetes type 2, Diet, Environment, Intervention, Implementation, Food stores, Supermarkets

## Abstract

**Background:**

Due to their central position in the modern food system, food stores present a unique opportunity to promote healthy dietary behaviour. However, there is a lack of insight into the factors that impede or enhance the implementation of nutritional interventions in food stores. We applied a systems innovation and implementation science framework to the identification of such barriers and facilitators.

**Methods:**

We conducted a systematic literature review. A search string was developed to identify qualitative and quantitative articles on environmental nutritional interventions in the food store. Four databases were systematically searched for studies published between 2000 and 2018. Eligible publications described study designs or original studies, focused on stimulating healthier dietary behaviour through environmental changes in retail settings and contained information on the perceptions or experiences of retailers or interventionists regarding the implementation process of the intervention. Context-descriptive data was extracted and a quality assessment was performed.

**Results:**

We included 41 articles, of which the majority was conducted in the USA and involved single stores or a mix of single and multi-store organisations. We categorized barriers and facilitators into 18 themes, under five domains. In the ‘outer setting’ domain, most factors related to consumers’ preferences and demands, and the challenge of establishing a supply of healthy products. In the ‘inner setting’ domain, these related to conflicting values regarding health promotion and commercial viability, store lay-out, (insufficient) knowledge and work capacity, and routines regarding waste avoidance and product stocking. In the ‘actors’ domain, no major themes were found. For the ‘intervention ‘domain’, most related to intervention-context fit, money and resource provision, material quality, and the trade-offs between commercial costs and risks versus commercial and health benefits. For the ‘process’ domain, most factors related to continuous engagement and strong relationships.

**Conclusions:**

This review provides a comprehensive overview of barriers and facilitators to be taken into account when implementing nutritional interventions in food stores. Furthermore, we propose a novel perspective on implementation as the alignment of intervention and retail interests, and a corresponding approach to intervention design which may help avoid barriers, and leverage facilitators.

**Trial registration:**

PROSPERO; CRD42018095317.

## Introduction

Various non-communicable diseases, including cardiovascular diseases (CVDs) and type 2 diabetes mellitus (T2DM) are attributable to unhealthy diets [1]. The promotion of healthy diets is therefore of utmost importance. Dietary behaviour is influenced by personal and environmental factors [2], among these, the local food environment plays a major role [3]. This environment comprises a ‘community environment’, describing available food sources at the community level, and a ‘consumer environment’, describing product offer, presentation, and pricing per source [3, 4]. In the USA, similar to observed in other Western countries, stores are the primary community environment food sources [5]. Furthermore, the literature supports that the consumer environment has substantial influence on dietary behaviour [6]. Therefore, the consumer environment in food stores represents a major point-of-influence on dietary behaviour, and thus an opportunity for dietary interventions [7].

Components for healthy food-store interventions (HFIs) aim to encourage healthier purchases, or discourage unhealthy ones. Common types are: 1) reducing or increasing product prices, 2) changing product availability, 3) multimedia promotion and advertisement for healthy products, and 4) providing product information at the point-of-purchase (POP) [8]. Correct implementation of these components is vital to intervention success.

The current evidence base on HFIs is dominated by a focus on intervention impact and process evaluation, but barriers or facilitators for implementation are generally not discussed in depth. The lack of a structured overview of such barriers and facilitators limits interventionists (people in health promotion positions) in the optimization of implementation processes. To address this gap in the literature, we conducted a systematic literature review that addresses the following research question: “What factors are reported in the literature which can present a barrier or facilitator to the implementation of HFIs?”

## Methods

We conducted the systematic literature review in accordance with the Preferred Reporting Items for Systematic Reviews and Meta-Analysis (PRISMA) protocol [9]. A systems innovation and implementation science framework was applied to categorize reported barriers and facilitators. The protocol was registered on PROSPERO under ID CRD42018095317. The study is a part of the SUPREME NUDGE project [10].

### Search strategy

First, the topic and terminology of HFIs were explored formatively. Next, a syntax was developed around three concepts: 1) food stores, 2) HFIs, and 3) intervention processes (development and implementation) and/or their evaluation. Various iterations were tested to assess the value of synonyms and exclusion terms. The full syntax can be found in Additional file 1.

Only publications from peer-reviewed scientific journals, in English, after January 1st 2000, were eligible for inclusion. This date was taken as a cut-off point to ensure relevance to the modern food store context, and supported by the lack of relevant studies from earlier years encountered during preliminary searches.

#### Selection criteria

We formulated five selection criteria:

1. The publication should describe an original study or design, in a full-length article.
2. The subject should be an intervention to stimulate healthier dietary behaviour through environmental changes, referring to adjustments in price, availability, promotion, or given POP (point-of-purchase) information, with the aim to promote healthier dietary behaviour [8], or formative research for such an intervention. This definition was based on a systematic review on interventions to promote healthier dietary behaviour, and thus closely fits our study focus.
3. The setting should be a food store with a fixed location and organisational structure. Restaurants, markets, stores set up/operated by interventionists, and take-outs were excluded, as these differ substantially from classic food-stores, as context, and thus fall outside the study scope. Stores were classified as ‘single stores’, or ‘multi-store organisations’.
4. The publication should discuss perceptions or experiences from retailers or interventionists regarding the intervention implementation process, in the results, discussion, or design (design papers only) section.
5. The intervention should be (at least partially) carried out by people from the food store organisation, or planned to be, in case of formative research/design publications.

#### Selection process

We conducted a search across 4 databases (PubMed, Embase, Web of Science and Scopus) on April 17th 2018. Duplicates were identified and removed by the first author (CNHM). Two authors (CNHM, JDM) independently screened entries based on title and abstract. Disagreements in the selection were discussed and resolved. Subsequently, CNHM performed a full-text assessment, with JDM independently repeating 10 % for validation. Reviews were excluded, but the reference lists of reviews and included publications were used for snowballing purposes by CNHM.

### Data extraction

CNHM extracted data on: author names, study type and objective, collected data, setting, the name, components and length of the intervention, reported intervention outcomes, and sections which described barrier and facilitators. Setting was classified by country, and as single, multi-store organisation, or mixed stores. Components were classified as utilising availability (making more healthy products available in a store), pricing (reducing prices to promote healthier products), promotion (using various media or games to promote the purchase of healthy products), or point-of-purchase information (demonstrations, tastings, or signage which highlight or promote healthy products) [8]. Intervention outcomes (health effects, sales effects, or process measures) were summarized and classified as either ‘substantial’ (process measures described by authors as ‘moderate’ or ‘high’, observed impact described as significant, or statistically significant findings) or ‘non-substantial’ (all other cases).

### Quality Assesment

Quality assement was conducted on the study level, by CNHM, with the QualSyst tool for systematic reviews [11]. This tool is suitable for qualitative and quantitative publications, both of which are included in this review. QualSyst uses criteria for the reporting of study objectives, design, methods, analysis, results, conclusions, and reflexions (for qualitative studies). The assessment of these criteria corresponds with a quality score: WEAK (score 0–0.59), MODERATE (0.60–0.79) or STRONG (0.80–1.00). As this study combines subjective evidence with objective data, we decided that an overall assessment of the strength of the body of evidence would be unrepresentative of the range of data utilized.

### Analysis

We used thematic synthesis to summarize the findings of this systematic literature review, aided by Atlas.ti software. Thematic synthesis is a commonly used method to synthesize qualitative research [12–15] and is based on the concept of thematic analysis [16]. We commenced with the open coding of relevant passages in the ‘results’ and ‘discussion’ sections of publications. After every ten articles, codes were re-examined and divided into sub-codes, if necessary. The codes were structured in a hierarchical tree: the passage-based codes were categorized under a hierarchy of one or more levels of broader codes, which in turn were nested under the domains of a theoretical framework. The open coding and use of a theoretical framework allowed for a combination of inductive and deductive research, although the broad nature of the theoretical framework allowed for all codes to fit under the theoretical domains. To synthesise (sub)themes, the passages under each code were summarized to describe the barriers or facilitators represented by this code, and the overarching (sub)themes. These barriers and facilitators were cross-referenced with three study characteristics: 1) the size of involved stores (single or multi-store organisations), 2) intervention outcomes (health behavioural, business-related, and process-related), and 3) quality score, where findings only supported by WEAK studies were marked in the overview table and excluded in the synthesis. Finally, (sub)themes were synthesised into a narrative.

#### Theoretical framework

Our theoretical framework is a combination of two models from separate fields: the Consolidated Framework For Implementation Research (CFIR) [17], from implementation science, and the constellation perspective [18], from systems innovation theory.

The CFIR classifies factors which influence implementation processes, assigning them to one of five domains-of-origin: 1) the outer setting (outside the implementation setting), 2) the inner setting (where the intervention is implemented), and characteristics of the 3) intervention, 4) process, and 5) involved individuals [17]. This model allows us to describe the general origin of barriers and facilitators.

The constellation perspective describes the inner working of socio-technological systems (e.g. food-store organisations). It conceptualises such systems as ‘constellations’ of three elements: culture (values, beliefs), structure (boundaries, rules, resources), and practices (activities, actions). Where culture and structure guide the development of practices by actors (people in the system), and carrying out these practices reinforces the culture and structure (a phenomenon called duality of structure). The social process through which constellations are (re)produced by actors is referred to as structuration, which manifests itself as the rigid ‘identity’ of constellations through their culture and structure [18, 19]. This model facilitates classification of barriers and facilitators within a food-store organisation, which we assumed is where many such factors originate for HFIs.

**Fig. 1:**
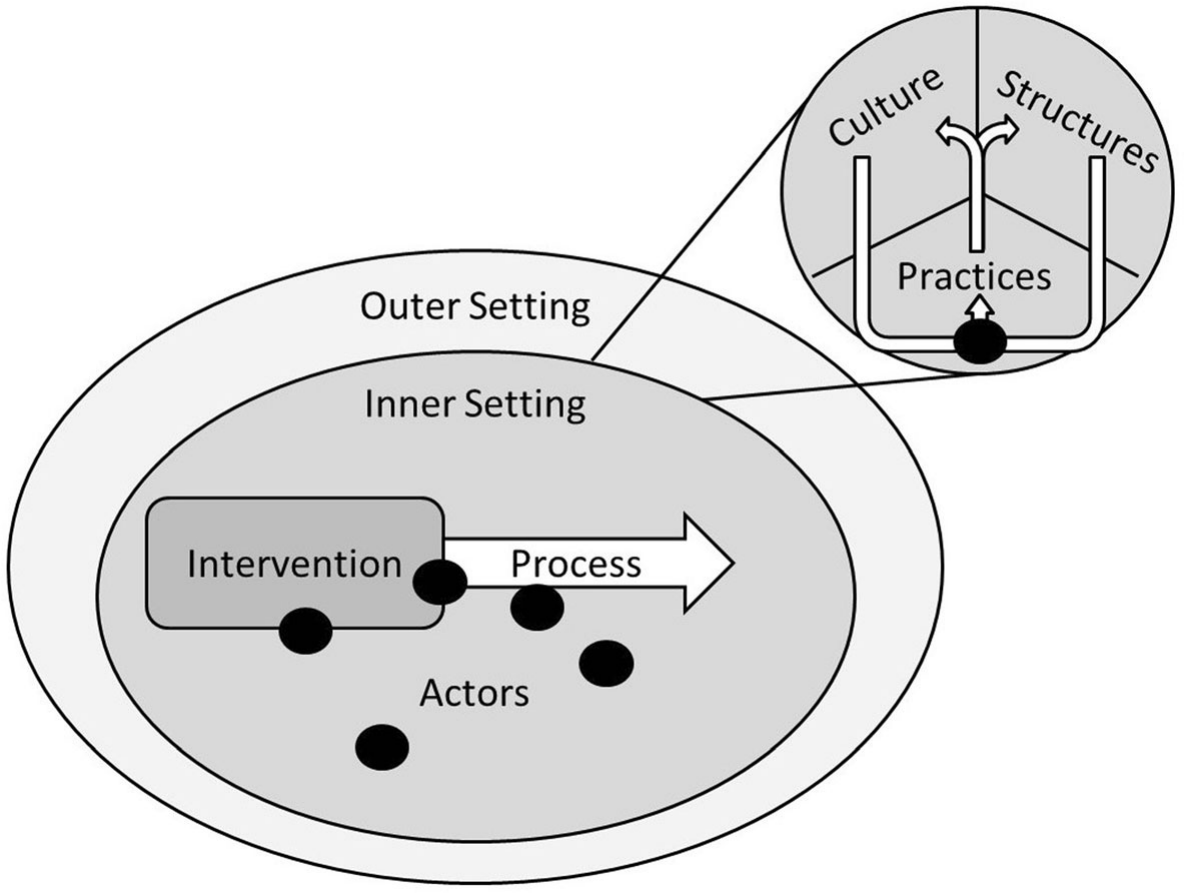
The theoretical framework. The inner setting of the CFIR is defined as a constellation of culture, structures, and practices. Its ‘individuals’ concept is redefined as actors, as used in the constellation perspective

We combined these models by conceptualising the inner setting of the CFIR as a constellation, and equating the ‘individuals’ from the CFIR to the ‘actors’ from the constellation perspective. In the resulting model, barriers and facilitators are categorized across five domains: the outer setting, (broad economic, social, and political forces which influence the intervention implementation process), the inner setting, (the food store organization context), and characteristics of the intervention, the implementation process, and the actors who operate the inner setting [17]. Barriers and facilitators in the inner setting are further categorized through three themes: the culture represents values, beliefs, and perspectives, which provide a general sense of priorities and goals for the constellation [18]. The *structure* represents physical, legal, financial and power structures in the constellation, which guide the activities in this constellation in the desired and reproducible directions [18]. The *practices* represent the translation of the culture and structure into the tangible activities, through which the inner setting achieves its goals [18]. The translation of the culture and structure into practices is carried out by the aforementioned *actors*. A visualisation of the model can be seen in Fig. 1.

#### Triangulation

To avoid bias, the analysis was triangulated between the authors after the initial coding (discussing the rationale behind selected passages and their assigned codes), after developing the hierarchical network (discussing the link between codes and their position in the hierarchy) and after the thematic synthesis (discussing the broader narrative).

## Results

**Fig. 2:**
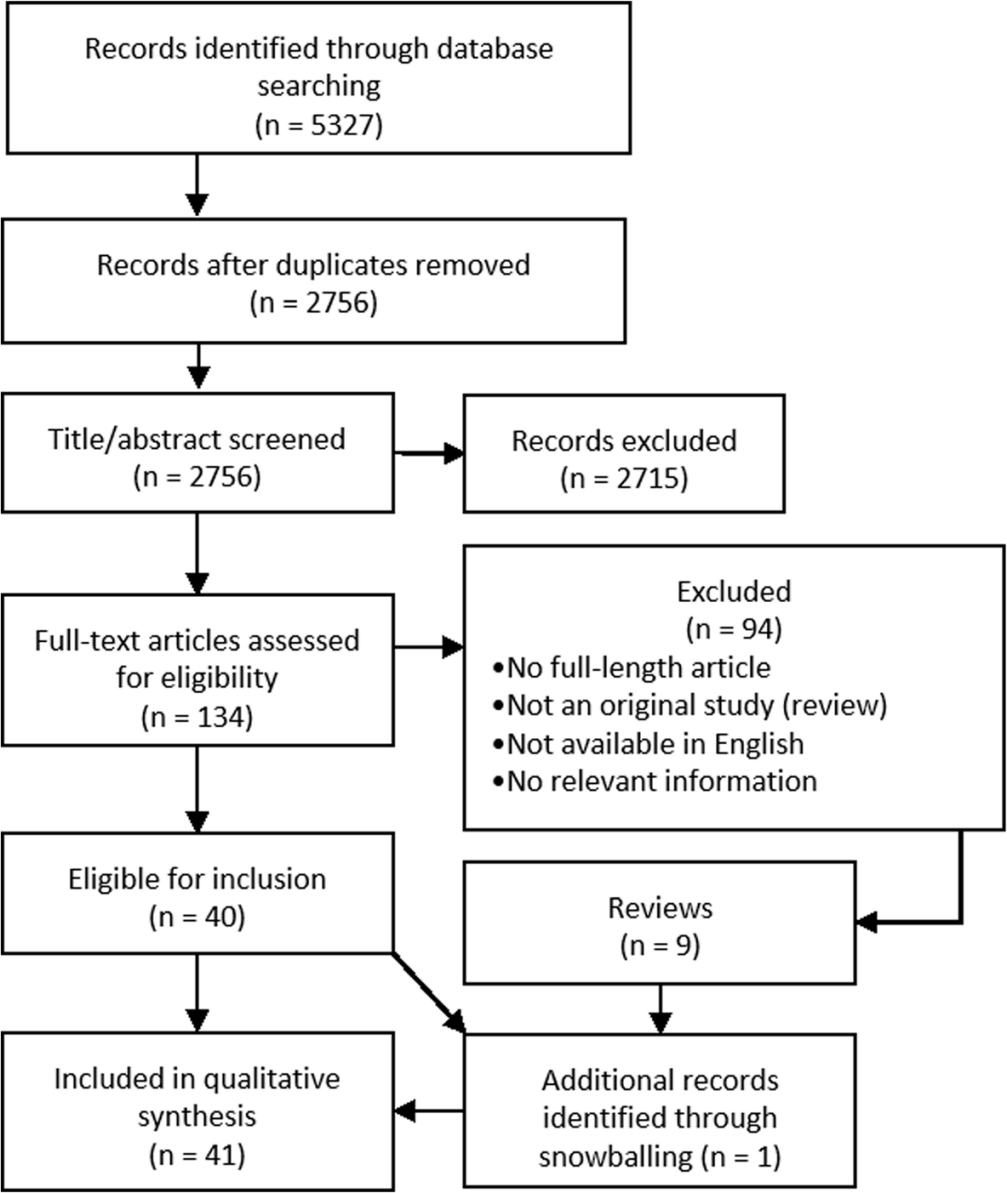
A flowchart of the publication selection process

A flowchart of the selection process and output is provided in Fig. 2. Our initial search yielded 5327 hits. Removal of duplicates left 2756 unique entries. After screening of titles and abstracts 134 publications remained. The full-text assessment selected 40 publications for inclusion, with a 100% agreement observed for the applied triangulation. Nine reviews were identified and used for snowballing, which yielded one additional publication. In conclusion, a total of 41 publications were included in the review.

Included studies were predominantly conducted in the US and mostly described process or outcome evaluations of HFIs. Out of 41 studies, 5 received a quality-assessment classification of WEAK, 22 of MODERATE, and 14 of STRONG [see Additional file 2 for all extracted study characteristics]. We identified 18 themes and 12 subthemes. Table 1 summarizes all identified barriers and facilitators, structured along the framework domains and their (sub)themes.

**Table 1:** This table summarizes the major (more than one source) identified barriers and facilitators, structured along their domain and (sub-)themes. Findings with major support (5+ sources, 12.1% of total) are marked in **bold**

Domains	Themes	(*Subthemes*) Main findings
Outer Setting	Product supply	− **Challenges in product supply** (20–33)^±+^, suppliers are unreliable (24, 30, 34, 35)^±+^_,_ geographic isolation (34, 35)^±^, demand fluctuations (30, 36)^±^
	Consumer characteristics	− **High demand unhealthy products** (20–24, 27–29, 31, 36–40)^±+^, **lower demand healthy products** (20, 23, 33, 36, 41)^±^, unhealthy products more profitable (20)^±^, **healthy perceived as expensive** (23, 24, 38, 40, 42)^±+^, customers prefer unhealthy products (23, 28)^±+^, **customers uninterested in health** (23, 36, 40, 42, 43)^±+^ and customers lack health knowledge (23, 42)^±+^
	Community relations	− Robberies and safety concerns (21, 40)^±^ + **Strong retailer-community relations** (23, 27, 31, 40, 41, 46)^±+^
	Competition	− Competitors steal customers (36, 37, 41)^±+^ + Lack of competition facilitates success (31, 35, 41)^±^
	Legislation	− Governmental taxes (59)^±^ + Health promotion legislation (46)^+^
	Media	− Stocking follows media exposure (29, 34)^±^
Inner Setting	Culture	*(Commerce)* − **Commercial interests** (26–28, 30, 32, 33, 48–50) + **Open for innovation and experimentation** (24, 26, 31, 33, 47, 51)^±+^ *(Health promotion)* − **Not feeling responsibility for community health** (23, 27, 28, 31, 33)^±+^, no affinity with health promotion (21, 31)^±^ + **Feeling responsibility for community health** (20, 23, 24, 27, 28, 33, 35, 42, 43, 47, 52)^±+^, affinity with health promotion (27, 47)^±+^
	Structure	*(Physical)* − **Space constraints** (27, 37, 40, 42, 45, 48, 49)^±+^, **limited storage/cooling facilities** (21, 22, 28, 29, 34–36, 38, 40)^±+^, **store renovations** (24, 26, 27, 43, 50)^±+^ *(Operational)* − **Unhealthy products restocked by suppliers** (21, 40, 44, 49, 54)^±+^, inconsistent product stocking (24, 44, 53)^±+^, supplier contracts (22, 38, 45)^±+^, difficulties returning unsold products (17, 34)^±^, constraints set by retailer (21, 27, 28, 49)^±+^, campaigns (42, 45, 50)^+^ *(Financial)* − **Products go to waste** (20, 22, 28, 30, 33–36, 40, 41, 44, 52)^±+^, limited financial resources (42, 47)^+^ *(Knowledge and capacity)* − **Lack relevant expertise** (23, 24, 31, 36, 42, 44, 47)^±+^, **limited time** (23, 31, 42–45, 48, 50)^±+^, **staff turnover** (26, 39, 50, 53)^±+^ + **Applicable business experience** (23, 28, 31, 47, 51)^±+^
	Practices	− **Stock in small quantities** (20, 22, 25, 26, 31, 33, 34, 37, 39, 40, 55)^±+^ + Flexible in establishing supply (30, 39)^±+^, waste limitation tactics (20, 31, 41)^±^
Actors	Personality traits	+ Pragmatism (31, 42, 51, 52)^±+^, desire to help (42, 47, 51)^±+^, tenacity (31, 51)^±^
	Psychological reactions	− Frustrations regarding intervention (29, 41, 42)^±+^, psychological stress (27, 39, 56)^±+^ + satisfaction from positive feedback (25, 31)^±^
Intervention	General characteristics	*(context-intervention fit)* − **Does not fit the context** (28, 42, 45–47)^+^. + **Fits the context** (27, 28, 37, 39, 42, 47, 48, 57)^±+^, **fits customer needs** (24, 37, 41, 45, 47)^±+^, fits retailer needs (37, 47)^±+^ *(flexibility)* − Inflexible design (27, 37, 42)^±+^ + **Adaption to context** (30, 35, 37, 48, 52, 53)^±+^, awareness of context complexity (27, 57)^±+^ *(complexity)* + Simple to implement (42, 47)^+^
	Components of the intervention	*(support)* − Difficulties maintaining provided equipment (29, 48)^±^, lack of intervention support (42, 52)^±+^ + **Financing start-up and running costs** (20, 22, 29, 35, 37, 44, 48, 52)^±+^, **provide promotion materials** (28, 29, 42, 47, 52)^±+^, monetary incentives (27, 37)^±+^ + **Building retailer-supplier relationships** (22, 26, 37, 40, 49, 55)^±+^, **subsidising stocking of products** (20–22, 27, 31, 39, 49, 55, 56)^±+^ + **Consultation regarding health promotion and business skills** (24, 29, 35–37, 42)^±+^, staff training (28, 35, 43)^±+^ *(promotion)* − Faulty placement materials (28, 52)^±^, retailers refuse negative promotion (28, 45)^+^, insufficient (re)supply of materials (42, 52)^±+^, materials lack durability (45, 52)^±+^ + **High quality materials** (24, 28, 42, 45, 47, 48)^±+^ (staff training) + **I**mproved engagement staff (35, 37)^±+^, improved skills for implementation intervention (29, 35, 48)^±^ (customer education) + Regarded as vital by retailers (20, 22, 37, 40)^±+^, improved demand promoted products (22, 37)^±+^ *(pricing)* − Regarded as unviable and potential risk (23, 49)^±+^
	Costs and benefits	*(costs and risks)* − **High running-costs** (21–23, 28–30, 33, 35, 37, 39–42, 44, 47, 49, 59)^±+^, high initial investment (22, 44)^±^, **substantial time investment** (23, 28, 29, 31, 42, 44, 53)^±+^, **substantial effort or impractical** (28, 30, 35, 42, 45)^±+^, **commercial risks** (21, 23, 28, 33, 37, 39, 56, 58, 59)^±+^ + **Low or minimal effort** (24, 27, 28, 45)^±+^ *(commercial benefits)* − **Commercial benefits do not outweigh risks and costs** (22, 28, 30)^+^ + **Increased profits and sales** (22, 24, 26, 28–30, 45, 47, 55)^±+^, **more customers** (22, 28, 30, 55)^±+^, **improved customer satisfaction** (28, 30, 45)^±+^, **improved public image** (28, 31, 45, 47, 55)^±+^, **establishment of partnerships** (35, 37, 47, 57)^±+^, general “positive outcomes” (27, 47)^±+^ (health benefits) − **Doubts regarding changing customer behaviour** (24, 27, 33, 42, 52)^±+^, lack of observable impact (35, 45)^±+^, loss of momentum (31, 45, 52)^±+^ + **Health promotion is inherently valuable** (20, 23, 24, 27, 33, 35, 42, 43, 47, 52)^±+^, **visible impact on sales and people** (24, 28, 29, 35, 45, 47)^±+^
Process	Engagement	− Unmotivated retailer (44, 52)^±^ + **Commitment and support from retailer** (24, 25, 27, 31, 42, 50, 60)^±+^, **retail-specific engagement strategies** (22, 24, 37, 45, 48)^±+^, providing staff training (35, 37)^±+^, build engagement incrementally (35–37)^±+^, develop intervention ownership (35, 37)^±+^, culture and language sensitive approach (26, 27, 37)^±^
	Collaboration	− Collaboration with competitors (28, 42)^+^ + **Good relationships collaborators** (21, 25, 26, 42, 48, 49)^±+^, **collaborative planning intervention** (27, 37, 42, 44, 46)^±+^, intervention helped developing collaborations (37, 47)^±+^
	Communication	− **Poor communication between collaborators** (31, 42, 45, 47, 50, 52)^±+^, lack of clarity on goals and agreements (21, 47, 52)^±+^, language and cultural barriers (21, 27, 30, 39)^±+^ + Clear communication (26, 27, 35, 42, 52)^±+^
	Organisation of activities	− Lacking planning and guidelines (42, 47, 52)^+^ + Thorough planning and transparency (42, 51, 53)^±+^

*Bullet point:*

−: factors interpreted as barriers

+: factors interpreted as facilitators

●: factors interpreted as both barriers and facilitators

*Superscript:*

±: supported by studies conducted among single stores

+: supported by studies conducted among multi-store organisations

### Outer setting

The outer setting represents the environment outside the intervention food-store. Identified themes were: product supply, consumer characteristics, community relations, competition, legislation, and media.

#### Product supply

The supply of intervention-promoted products was usually discussed as a barrier. Most frequently, single retailers perceived challenges in maintaining a constant supply of intervention products, mainly related to difficulties in finding a supplier [20–33]. In some cases, they perceived unreliability of the suppliers as the cause [24, 30, 34, 35], whereas for two studies in remote areas geographic isolation was the issue [34, 35]. Some single-store retailers perceived fluctuations in demand as a barrier for determining how much product needed to be stocked [30, 36]. These issues were primarily encountered by single-store retailers, who lack robust supply structures.

#### Consumer characteristics

Consumer characteristics mainly presented barriers. Frequently, retailers perceived a higher demand for unhealthy than for healthy products [20–24, 27–29, 31, 33, 36–41], and thus regarded unhealthy products as more profitable [20]. They often attributed this to customers perceiving healthy products as expensive [23, 24, 38, 40, 42], personal habits [23, 28] or taste preferences [23]. In one case, a declining customer base led to decreasing demand [31]. Retailers often believed that consumers lacked interest in [23, 36, 40, 42, 43], and knowledge of [23, 42] health. However, exceptions were reported where customers were interested in healthy products [23], demand was at profitable levels [44], and greater interest in health was perceived [45]. Such a shift towards greater interest in health was regarded by some as a prerequisite for effective health promotion [40].

#### Community relations

Community relations seemed most relevant for single stores. Interventionists frequently perceived strong community relationships as potential motivators for retailers [23, 27, 31, 40], or a way to gather community support [41, 46]. In two cases, retailers perceived robberies as a threat to their own and interventionists’ safety [21, 40]; turning the community into a barrier.

#### Competition

Multi-store competitors were perceived as a barrier, as they could offer promoted products for better prices [36, 41], or present an alternative for dissatisfied customers [37], leading to customer loss. Furthermore, some single-store retailers believed a lack of competition to contribute to intervention success [31, 35, 41]. Instability in the competitive environment seemed to deter single-store retailers from participation [43]. In contrast, multi-store retailers said they kept an eye on their competitors, and might copy them in terms of product offer [28] or intervention participation [47].

#### Legislation

Legislation was discussed little. In one study, the interventionists perceived governmental legislation as a potential facilitator for the implementation of interventions [46].

#### Media

Media was discussed little. In two cases, single-store retailers said they adjust their offer to what is promoted in the media [29, 34].

### Inner setting

The inner setting refers to the food-store organisation where interventions are implemented. Identified (sub) themes were: *culture*, (commercial and health values), *structure* (physical, operational, financial, and knowledge structures), and *practices* (stocking and waste management).

#### Culture

Two cultural factors were identified; commercial-and health values, both appearing with high frequency. Prioritisation of commercial interests was described as a major determinant in retailers’ organisational decision making [26, 28, 30, 32, 33, 48]. Conflict between commercial interests and intervention interests sometimes presented a barrier [27, 28, 49, 50]. In various other cases, food stores exhibited a culture of innovation and experimentation, possibly making them more open to interventions [24, 26, 31, 33, 47, 51].

Often, organisations valued health (e.g., recurring involvement in health promotion), which resulted in a common interest of retailers and interventionists. Retailers frequently showed responsibility for [20, 23, 24, 27, 30, 33, 35, 40, 42, 43, 47, 52] and awareness of [23, 27, 33] the health of their community. If there were strong c*ommunity relations*, as discussed earlier, health values might thus form a strong motivator for implementation. In some cases retailers were already experienced in community and health activities [27, 47]. Among multi-store retailers, such affinity with the subject was attributed to organisational appreciation of health [47] or perceived expectations from customers [47]. In contrast, some retailers considered health promotion as not their responsibility [23, 28, 31, 33], which possibly reduced confidence in and success of interventions [21, 23, 27, 31].

#### Structure

Structures were often described as important barriers. Physical structures were most frequently discussed: many retailers lacked the necessary storage facilities to stock fresh products promoted by the intervention [21, 22, 28, 29, 34–36, 38, 40], and available space to display promotional materials was limited [37, 42, 45, 48, 49], whereas retailers with more space seemed more supportive of the intervention [27]. In one study, multi-store retailers would present posters in frames on the wall, which varied in size and required posters of specific dimensions [45]. Store renovations often disrupted the implementation process [24, 26, 27, 43, 50], and one single-store intervention was limited by the store lay-out [40].

Operational structures were less common: some single-store interventions were constrained by promotional campaigns [42, 45, 50] and contractual obligations regarding product placement and stocking [22, 38, 45]. In several other studies, retailers themselves would place constraints on intervention activities (e.g. space use, when to approach customers) [21, 27, 28, 49]. Furthermore, single-store retailers expected returning unsold (fresh) products to the wholesaler to be impossible [30, 40], which was a barrier to stocking these. Finally, the restocking of products often lacked a consistent structure among single-store retailers [24, 44, 53]. An exception to this was when external parties did the restocking of (usually unhealthy) products [21, 40, 44, 49, 54]. Large-scale stocking was perceived as a facilitator which could limit disruptions in the process [31].

Financial structures were discussed in two forms. First, the waste of fresh products (and associated financial losses) was a frequent and major barrier among single-store retailers [20, 22, 28, 30, 33–36, 40, 41, 44, 52]. Some studies discussed how a lack of financial resources was perceived as a minor barrier, whereas having such resources was considered a facilitator [42, 47]. These financial issues might be related to the emphasis placed in commercial values, discussed under *culture*.

Multiple studies discussed capacity as a structural element: Lack of organisational expertise on health promotion activities was a frequently perceived barrier [23, 24, 31, 36, 42, 44, 47], as was a lack of available man-hours [23, 31, 42–45, 48, 50]. Capacity was sometimes limited further by high staff turnover rates [26, 39, 50, 53], which made it difficult to train them. However, retailers often had business experiences they could apply to the benefit of the intervention [23, 28, 31, 47, 51].

#### Practices

Practices related to product stocking and waste management. Due to the aforementioned issue of food waste and cultural prioritisation of profitability, single-store retailers usually stock fresh products in single, financially inefficient, quantities, to avoid waste [20, 22, 25, 26, 31, 33, 34, 37, 39, 40, 55]. This frequently presented a barrier to interventions aimed at stocking more such products. Some retailers resolved this problem through stocking frozen foods [20, 41], or using near-expired products in daily offerings [31]. Furthermore, interventionists considered the flexibility of single-store retailers in establishing new supply lines a facilitator for stocking new products [30, 39].

### Actors

Actors incorporate an intervention into their regular practices. Certain personality traits among these actors, the retailers, were considered facilitators for implementation. These were a pragmatic [31, 51, 52], tenacious [31, 51], accommodating [42], empathic [47, 51], or philanthropic [42] character (in line with the facilitative culture values discussed earlier), or striving to address a perceived need [51]. Furthermore, actors’ psychological reactions to the intervention can present barriers, when perceived risks cause psychological distress [27, 39, 56], or, set-backs and problems in the process lead to frustration [29, 41, 42]. Reactions can also present a facilitator, such as when retailers experience satisfaction from positive customer feedback on the intervention [25, 31].

### Intervention

The intervention domain encompasses everything directly related to the intervention. Identified (sub)themes were: general characteristics (context-intervention fit, flexibility, and complexity), components (support, promotion, staff training, customer education, and pricing), and *costs and benefits* (costs and risks, commercial benefits, and health benefits).

#### General characteristics

The most frequently discussed general characteristic was ‘context-intervention fit’: how closely the intervention fits the food-store context in terms of culture, structure, and practices. This is naturally intertwined with the barriers and facilitators in the Internal setting domain. Various studies note the facilitative properties of shared values [28, 42, 47, 57] and target groups [27, 47], integrating the intervention in existing organisational practices [37, 39, 47, 48], and intervention design fitting the store layout [27]. Multiple studies illustrate the value of aligning the intervention with the needs of retailers [37, 47] or their customers [24, 37, 41, 45, 47]. In cases where the intervention did not fit with the practices, facilities [45], target group [28, 46, 47], or personalities [42, 47] of retail organisations, this was considered a barrier.

A related, though less discussed, subtheme is intervention flexibility. When an intervention could be adapted to the context [35, 37, 48, 53], sometimes independently by retailers [30, 35, 52], this was regarded as a facilitator. This might require interventionists to be aware of the complexity of the implementation context, also a perceived facilitator [27, 57]. In some cases, difficulties in adapting the intervention [37], tight regulations [42], or ignored requests from retailers [27] were perceived barriers.

The final subtheme is complexity. In two studies, multi-store retailers considered the intervention simple to implement [42, 47], whereas in another case an intervention was considered (needlessly) complex due to issues in project management, lack of required knowledge, and perceived low relevance [47], or an overabundance of activities [42]. One time, collaborators (including multi-store retailers) felt the intervention should have been more complex, as they felt it insufficiently addressed social determinants [42].

#### Components of the intervention

Components include behaviour change elements aimed at the target group, as well as supportive elements aimed at retailers. Most discussed were supportive activities. Retailers were often supported through the provision of physical resources: refrigeration or display equipment [35, 37, 44, 48, 52], and promotional materials [28, 29, 42, 47, 52], and sometimes financial resources: monetary incentives [27, 37], subsidies for electricity [20], start-up costs [22], time costs [29], and ‘general resources’ [43]. Such resources might help resolve previously discussed *structural* barriers. Assistance in establishing supply lines, by negotiating vendor prices, facilitating vendor-retailer relationships [22, 26, 37, 40, 49, 55], or providing subsidies, coupons, or samples for specific products [20–22, 27, 31, 39, 49, 55, 56] was often considered a facilitator, and could present a solution to the previously discussed product supply barriers. Another common support type focussed on human capacity, such as providing of expertise regarding health promotion and business skills [24, 29, 35–37, 42], staff training [28, 35, 43], and providing support staff [42]. These activities might help resolve barriers regarding human capacity, as discussed under structure. Sometimes, support activities failed due to retailers not acting on the provided advice [44]. A lack of support activities was sometimes named as a barrier [42, 52].

Second-most discussed were promotion activities. Retailers often emphasized that promotional materials should be of high quality [24, 28, 42, 45, 47, 48], and free for customers [45]. Noted barriers were multi-store retailers opposing negative ‘promotion’ for unhealthy products [28, 45], likely due to commercial interests or contracts, and retailers regarding the physical quality [45, 52] or resupply of materials as lacking [42, 52]. Interventionists were sometimes concerned about the wrongful use of promotional materials by single-store retailers [28, 37, 52].

Training, education, and pricing were discussed little. Staff-training, was perceived to improve engagement [35, 37], and improve intervention implementation [29, 35, 48]. Engaging part-time staff was considered challenging [35]. Customer education was sometimes considered as a facilitator [20, 22, 37, 40], as it improved the demand for intervention-promoted products [22, 37], the lack of which was a consumer characteristics barrier. One study found that in single stores, fluctuating supplier prices and a single product-range presented barriers to pricing components [23].

#### Costs and benefits

The perceived balance between costs, risks, and benefits, influences retailers’ support for an intervention. The perception of this balance is likely to influence whether *commercial values* present a barrier. The most frequently perceived costs were time (e.g. planning and implementation [23, 28, 29, 31, 42, 44, 53]), effort (especially for impractical components [28, 30, 35, 42, 45]), and financial investments (e.g. start-up and running costs [21–23, 28–30, 35, 39–42, 44, 47, 58, 59]), with space being less common [28]. Frequently noted risks were the intervention disrupting regular business and driving customers away [23, 28, 33, 37, 39, 56, 58, 59], or facilitating theft [21, 37, 39]. Low intervention costs, in terms of effort, were perceived as a facilitating factor [24, 27, 28, 45]. One study proposed that interventions of limited duration might be more easily implemented [28]. Various of these costs and risks might be alleviated through the discussed supportive *Intervention components*.

The benefits of interventions can be split into commercial benefits and health benefits, which can be linked back to the values of the same name, discussed under culture. Retailers frequently perceived commercial benefits: improvements in sales [22, 24, 26, 28–30, 45, 47, 55], number of customers [22, 28, 30, 55], customer relations [28, 30, 45], market position and visibility [28, 31, 45, 47, 55], opportunities for network building [35, 37, 47, 57], and undefined ‘positive outcomes’ [27, 47].

In many cases retailers expressed appreciation of the health improvement goals of interventions [20, 23, 24, 27, 33, 35, 42, 43, 47, 52]. This appreciation is based on the perceived effectiveness of the intervention, and thus, retailers considered visible impact among customers [24, 29, 35, 45] or their staff [28, 35, 47] a facilitator, as it reinforced trust in the intervention [30]. Unfortunately, in various cases retailers were not convinced the intervention could improve health [24, 27, 33, 42, 52], and were demotivated by a lack of observable impact [31, 35, 45, 52].

### Process

The process of an intervention refers to the collaborative activities towards implementing the intervention. Identified themes were: engagemen*t*, *collaboration*, *communication*, and *organisation of activities*.

#### Engagement

This theme relates to recruiting collaborators, and keeping them involved and committed. Engaging retailers to build commitment and support frequently perceived as a facilitator for success [24, 25, 27, 31, 42, 50, 60]. Perceived methods were: recurring contact and interaction with retailers [37], providing retailers with educational materials on the intervention [37, 45], providing staff training [35, 37], demonstrating a demand for healthy products [24](potentially a solution to the barriers discussed under consumer characteristics), and using other intervention stores as examples for the value of the intervention [22]. Sufficient engagement was perceived to create feelings of ownership among retailers and staff [35, 37], which might improve commitment. Some studies argued for engagement in incremental steps [35–37], and being sensitive to the culture and language of retailers [26, 27, 37]. Encountered barriers to engagement were: retailers who were forced to participate by superiors [52], and declining motivations when intervention impact seemed low [44], likely because this lowered perceived benefits. A lack of support from the retailers for the intervention strategy was a barrier in one study [42].

#### Collaboration

This theme refers to the collaboration between interventionists and retailers. Good relationships between the collaborators was a frequently noted facilitator [21, 25, 26, 42, 48, 49]. Co-creation of the intervention was often perceived to help avoid contextual barriers, improving the context-intervention fit, and stimulate feelings of ownership among the retailers [27, 37, 42, 44, 46]. In one study, multi-store retailers regarded the involvement of non-retail partners as valuable [47], whereas some single-store retailers regarded the lack of other involved societal actors as a constraint [52]. In two studies, single-store retailers regarded the process as beneficial for the development of inter-retailer collaborations [37, 47], whereas some multi-store retailers regarded working with competitors as problematic [28, 42] possibly due to the issues with *competitors*, discussed under the *outer setting*.

#### Communication

When effective communication was realized, retailers regarded this as a strong facilitator for personal engagement, and intervention implementation [26, 35, 42]. This could involve the clear communication of intervention aims [52], guidelines [27], or structures to share and retain experiences [26]. In cases where communication was poor, this was perceived to constrain the intervention process and collaboration [42, 47, 50, 52], and perceived as a reason for loss of momentum [31, 45, 52]. Poor communication was experienced as lack of clarity regarding intervention goals [47, 52], intervention activities [21, 52], program policies to guide these activities [52], the advantages for retailers [21], and the distribution of responsibilities [52]. Among single-store retailers, collaboration between interventionists and retailers [21, 27, 39], or retailers and customers [27, 30], was sometimes perceived to be constrained by language and cultural barriers.

#### Organisation of activities

Organisation factors, such as thorough planning [53], structure [42] and transparent decision making [51], were sometimes named as facilitators. Other times, the intervention process was perceived to be constrained by shortcomings in task management [47], planning [47], or too strict timelines [42].

### Comparing barriers, facilitators, to study characteristics

Identified barriers and facilitators were cross-referenced with intervention outcomes, store size, and quality score.

#### Outcomes

A number of barriers and facilitators were generally identified in ‘successful’ interventions, meaning studies where health-behavioural, business-related, or process-measure outcomes were statistically significant in the expected direction, or described by authors as ‘moderate’/‘high’. These barriers were: high turnover among retail staff (4/4 studies [26, 39, 50, 53]**),** inconsistent product restocking (3/3 studies [24, 44, 53]), restocking of (unhealthy) products by outside parties (4/5 studies [21, 40, 44, 49, 54]), disruptions by store renovations (3/5 studies [24, 26, 27, 43, 50]), and the importance placed on commercial interests (3/6 studies [26, 28, 30, 32, 33, 48]). Facilitators were: a good relationship between retailers and interventionists (5/6 studies [21, 25, 26, 42, 48, 49]) and commitment and support from the retailer (4/7 studies [24, 25, 27, 31, 42, 50, 60]). There were no barriers or facilitators which were generally identified by ‘unsuccessful’ interventions, meaning studies with statistically non-significant, significant in an unexpected direction, or ‘low’ outcomes.

#### Size

Several factors were only encountered by studies among single-store retailers. Such barriers were: difficulties in predicting product demand for restocking [30, 36], and establishing and maintaining a stable supply [20–33], danger from robberies [21, 40], instability in the market discouraging participation [43], product stocking being based around media coverage [29, 34], limitations in store lay-out [40], conflicting pre-existing plans [42, 45, 50], contractual obligations for product placement and stocking [22, 38, 45], difficulties regarding the return of unsold products [30, 40], inconsistency in restocking [24, 44, 53], unhealthy products being consistently restocked by external parties [21, 40, 44, 49, 54] product waste [20, 22, 28, 30, 33–36, 40, 41, 44, 52], needing to stock fresh product in small inefficient quantities [20, 22, 25, 26, 31, 33, 34, 37, 39, 40, 55], potential wrongful use of promotional materials [28, 37, 52], fluctuating supplier prices [23], the financial unviability of multiple concurrent discounts [23], criticism on the lack of additional involved societal partners [52], and language and cultural barriers [21, 27, 30, 39]. Facilitators among single stores were: strong community relations [23, 27, 31, 40], the use of community engagement [41, 46], a lack of close competitors [31, 35, 41], and participation being beneficial for developing inter-retailer collaborations [37, 47].

Among multi-store retailers, encountered barriers were: display frames differing between organisations [45], criticism of intervention simplicity for its purpose [42], opposition to discrimination against unhealthy products [28, 45], and working with competitors being regarded as problematic [28, 42]. Facilitators were: a tendency to copy competitors in terms of product offer [28] but also intervention participation [47], valuing health promotion [47] or expectations from their clientele to do so [47], regarding intervention implementation as simple [42, 47], and valuing the involvement of non-retail partners [47].

#### Quality score

Two factors were only supported by studies with a WEAK quality score. These were store renovation being difficult due to rules set by the landlord [29] and irregular opening hours conflicting with intervention activities [49].

## Discussion

In this study we aimed to gain insight into barriers and facilitators to the implementation of HFIs, thus facilitating the utilisation of the consumer environment to promote healthier dietary behaviour [4]. Our findings were structured across five domains (outer setting, inner setting, actors, intervention, process), within which we identified 18 themes of barriers and facilitators, a summary of which can be found in Table 1. This overview facilitates interventionists in anticipating these barriers and facilitators, possibly improving the implementation of their interventions. Currently, one comparable review has been conducted, with a narrower focus on the ability and willingness of US store owners to use choice-architecture and marketing-mix strategies to encourage healthy consumer purchases [61]. Its findings seem comparable to our own. Our discussion aims to take the analysis a step further, by exploring how interventionists can apply our insights to implementation processes, and up-scaling.

### Aligning intervention and food store

Our findings illustrate a fundamental issue for the implementation of HFIs. From a systems innovation perspective, implementing a HFI can be conceptualised as embedding a smaller (intervention) constellation in a larger (food-store) constellation [62]. Both have their own culture, structures, and practices, serving a certain interest [18, 62]. From this perspective, barriers and facilitators for implementation might be present cases where the central interests (and accompanying culture, structures, and practices) of the intervention and food-store constellations misalign (barriers) or align (facilitators) [18].

Our findings illustrate that a primary interest of the food-store constellation is commercial viability (e.g., the importance of demand for products, retaining customers, limiting costs), whereas that of the intervention constellations arguably is the stimulation of healthier diets. When the promotion of health is served by actions perceived (by retailers) as commercially detrimental, the resulting conflict presents a barrier, as retailers likely oppose actions detrimental to their business. Examples would be interventionists seeking to decrease the sales of unhealthy products (likely leading to lower revenue) [28], or increasing the stocking of products perceived as low-in-demand (possibly leading to waste and losses) [49]. In contrast, facilitators, such as community engagement, and waste-avoidance strategies, combine commercial benefits (more customers, higher profits) with stimulating healthier diets. By working towards an alignment of interests, and resolving or avoiding misalignment, implementation processes might be facilitated.

However, interventions and food stores vary in their characteristics, and anticipating how (mis)alignment will manifest is challenging. A transition-management approach, where interventionists and retailers construct a shared agenda, integrating interests and ideas of both parties into a mutually-acceptable path forward, might overcome this [18, 63]. This agenda facilitates intervention design and implementation through its co-creative character: Enabling interventionists to utilise the contextual knowledge of retailers to develop intervention components with a strong fit to this context. Furthermore, retailers can utilise interventionists’ knowledge to increase their success in implementing intervention components [64].

Once an agenda detailing goals and strategies is established, these strategies must be implemented [18, 63]. For this goal, collaborating food-store constellation actors perform ‘experiments’ (intervention components) throughout the constellation. Meanwhile, the agenda and experiments are continuously adapted to new insights, in a process of reflexive learning. By spreading (successful) experiments through the constellation, change (intervention institutionalisation) is facilitated.

Interventionists should be aware that this method implies substantial flexibility in the intervention design. To maintain some continuity across multiple settings, interventionists might define ‘maximum-deviation boundaries’ for aspects of their intervention.

#### Considerations regarding implementation and upscaling

To achieve meaningful impact, interventionists often aim to upscale successful interventions to broader contexts, e.g. more stores or organisations. From our findings we hypothesize that the size of food-store organisation substantially influences the feasibility and potential of this endeavour. Single food-stores, as less established constellations, seem more flexible in their routines and boundaries [30, 39], likely due to lower levels of structuration [18, 62]. This facilitates innovation (e.g. implementing an intervention) [18, 62], as alignment is more easily achieved, and misalignment more easily resolved. However, this low level of structuration might cause institutionalisation of intervention elements in the organisation to be less robust, making the realization of lasting changes a challenge. Furthermore, these stores were limited to a single location, which limits potential up-scaling.

In contrast, multi-store organizations, being highly established constellations, likely have higher levels of structuration, which makes it initially challenging to achieve innovative changes [18, 62]. However, when intervention elements are accepted, this structuration facilitates their implementation, and retention over time [18, 62]. Furthermore, such robust structures form a tool for scaling up [18, 62], as each organisation has multiple stores, and institutionalised changes will likely be introduced throughout the organisation.

Clearly both sizes of organisations require fitting approaches for implementation and upscaling. Regarding single food-stores, interventionists should consider involving multiple organisations at once, to compensate for the limited up-scaling potential, or seek alternative avenues for dissemination (e.g. professional networks). In contrast, interventionists working with multi-store organizations should anticipate higher required effort for the initial development and implementation stage, whereas the up-scaling is less likely to present a problem if the intervention is perceived as successful.

### Strengths and limitations

A major strength is the number of included publications, and broad coverage in terms of contexts, study designs, and intervention designs. Despite this range, no contradictory findings were encountered, which strengthens our findings. Furthermore, the process of study selection and analysis was triangulated at multiple points, improving its scientific rigour.

The first limitation is that a major part of the included publications discussed barriers and facilitators primarily in the discussion section. As this section involves authors’ own interpretations and non-systematic observations, biases might be present. However, due to the limited number of studies which examined barriers and facilitators, we decided to accept this shortcoming. Second, studies among multi-store organizations are underrepresented in the sample. Therefore, conclusions regarding this group should be interpreted with caution. Third, only half of the included publications reported measures of the intervention process or impact. Thus, conclusions regarding trends between reported barriers and facilitators, and intervention-related measures should be interpreted with care.

## Conclusion

In conclusion, we conducted a systematic review of the literature on HFIs to develop an overview of barriers and facilitators for their implementation, structured across five domains (outer setting, inner setting, actors, intervention, process). Though this overview is informative, these factors should not be considered separately. Building on these results, we argued that an underlying mechanism of barriers and facilitators is the (mis)alignment of retailers’ and interventionists’ interests. We proposed how interventionists might develop their interventions to facilitate alignment of these interests. Finally, we discussed how interventionists might upscale their interventions more effectively. Through these insights, the implementation of HFIs might be further optimised, to contribute to the reduction of the global burden of non-communicable diseases.

### Research recommendations

Based on our findings we recommend future HFI study protocols to make reflection on the implementation process, its barriers and facilitators, and how this relates to intervention outcomes (if these are measured), a standardized part of evaluations, as structured reflections are currently rare. Furthermore, the role of actor characteristics on the implementation process of HFIs seems to be an underdeveloped topic. Additionally, though our overview is comprehensive, it lacks insight into prioritisation of the described barriers and facilitators, which should be explored in the future. Finally, we recommend interventionists to implement and evaluate the proposed transition management approach.

## Supplementary information

**Additional file 1.** Search Syntax

**Additional file 2.** Extracted Data

## Data Availability

All data generated or analysed during this study are included in this published article and its supplementary information files.

## Abbreviations

CFIR: Consolidated Framework For Implementation Research
CVDs: Cardiovascular diseases
HFI: Healthy food-store intervention
PRISMA: Preferred Reporting Items for Systematic Reviews and Meta-Analysis
T2DM: Type 2 Diabetes Mellitus
