# Supplementary material for: Systematic review: a systems innovation perspective on barriers and facilitators for the implementation of healthy food-store interventions
Source: Int J Behav Nutr Phys Act. 2019 Nov 21;16:108. doi: 10.1186/s12966-019-0867-5 (PMC6868845; doi:10.1186/s12966-019-0867-5)
Supplement: Supplementary file 1 — Additional file 1. Search Syntax [file 12966_2019_867_MOESM1_ESM.pdf]

Supplementary File 1: Search Syntax

PubMed

T1

((Supermarket*[Title/Abstract] OR store[Title/Abstract] OR stores[Title/Abstract] OR retail*[Title/Abstract] OR shop*[Title/Abstract])) NOT (pharmacy[Title/Abstract] OR pharmacies[Title/Abstract] OR pharmaceutical[Title/Abstract] OR pharmacist*[Title/Abstract] OR drug[Title/Abstract] OR medicine[Title/Abstract] OR tobacco[Title/Abstract] OR smoke[Title/Abstract] OR smoking[Title/Abstract] OR marihuana[Title/Abstract])

T2

((diet[Title/Abstract] OR dietary[Title/Abstract] OR nutrition[Title/Abstract] OR nutritional[Title/Abstract] OR food[Title/Abstract] OR foods[Title/Abstract])) AND (Intervention*[Title/Abstract] OR program[Title/Abstract] OR programs[Title/Abstract] OR programme*[Title/Abstract] OR project[Title/Abstract] OR projects[Title/Abstract] OR campaign[Title/Abstract] OR campaigns[Title/Abstract])

T3

(Barrier*[Text Word] OR challenge*[Text Word] OR facilitat*[Text Word] OR opportunit*[Text Word] OR evaluat*[Text Word] OR process[Text Word] OR implement*[Text Word] OR enhancing[Text Word] OR impeding[Text Word] OR constrain[Text Word] OR "formative research"[Text Word] OR "bottom-up approach"[Text Word] OR "bottom-up development"[Text Word] OR "participatory action research"[Text Word] OR "participatory research"[Text Word])

Full search

T1 AND T2 AND T3 Filters: Publication date from 2000/01/01

Web of Science (Core Collection)

T1

(TS=(Supermarket* OR store OR stores OR retail* OR shop*) NOT TS=(pharmacy OR pharmacies OR pharmaceutical OR pharmacist* OR drug OR medicine OR tobacco OR smoke OR smoking OR marihuana))

T2

(TS=(diet OR dietary OR nutrition OR nutritional OR food OR foods) AND TS=(Intervention* OR program OR programs OR programme* OR project OR projects OR campaign OR campaigns))

T3

(TS=(Barrier* OR challenge* OR facilitat* OR opportunit* OR evaluat* OR process OR implement* OR enhancing OR impeding OR constrain OR “formative research” OR “bottom-up approach” OR “bottom-up development” OR “participatory action research” OR “participatory research”))

Full search

T1 AND T2 AND T3 Timespan=2000-2018 Search language=English

Embase

T1

('supermarket*':ti,ab,kw OR 'store':ti,ab,kw OR 'stores':ti,ab,kw OR 'retail*':ti,ab,kw OR 'shop*':ti,ab,kw) NOT ('pharmacy':ti,ab,kw OR 'pharmacies':ti,ab,kw OR 'pharmaceutical':ti,ab,kw OR

'pharmacist*':ti,ab,kw OR 'drug':ti,ab,kw OR 'medicine':ti,ab,kw OR 'tobacco':ti,ab,kw OR 'smoke':ti,ab,kw OR 'smoking':ti,ab,kw OR 'marihuana':ti,ab,kw) AND [2000-2018]/py

T2
('diet':ti,ab,kw OR 'dietary':ti,ab,kw OR 'nutrition':ti,ab,kw OR 'nutritional':ti,ab,kw OR 'food':ti,ab,kw OR 'foods':ti,ab,kw) AND ('intervention*':ti,ab,kw OR 'program':ti,ab,kw OR 'programs':ti,ab,kw OR 'programme*':ti,ab,kw OR 'project':ti,ab,kw OR 'projects':ti,ab,kw OR 'campaign':ti,ab,kw OR 'campaigns':ti,ab,kw) AND [2000-2018]/py

T3
(barrier* OR challenge* OR facilitat* OR opportunit* OR evaluat* OR process OR implement* OR enhancing OR impeding OR constrain OR 'formative research' OR 'bottom-up approach' OR 'bottom-up development' OR 'participatory action research'/exp OR 'participatory action research' OR 'participatory research'/exp OR 'participatory research') AND [2000-2018]/py

Full search
T1 AND T2 AND T3 AND [2000-2018]/py

Scopus

T1
(TITLE-ABS-KEY(Supermarket* OR store OR stores OR retail* OR shop*) AND NOT TITLE-ABS-KEY(pharmacy OR pharmacies OR pharmaceutical OR pharmacist* OR drug OR medicine OR tobacco OR smoke OR smoking OR marihuana))

T2
(TITLE-ABS-KEY(diet OR dietary OR nutrition OR nutritional OR food OR foods) AND TITLE-ABS-KEY(Intervention* OR program OR programs OR programme* OR project OR projects OR campaign OR campaigns))

T3
(TITLE-ABS-KEY (Barrier* OR challenge* OR facilitat* OR opportunit* OR evaluat* OR process OR implement* OR enhancing OR impeding OR constrain OR "formative research" OR "bottom-up approach" OR "bottom-up development" OR "participatory action research" OR "participatory research"))

Full search
T1 AND T2 AND T3 AND PUBYEAR > 2000
