# Supplementary material for: Systematic review: a systems innovation perspective on barriers and facilitators for the implementation of healthy food-store interventions
Source: Int J Behav Nutr Phys Act. 2019 Nov 21;16:108. doi: 10.1186/s12966-019-0867-5 (PMC6868845; doi:10.1186/s12966-019-0867-5)
Supplement: Supplementary file 2 — Additional file 2. Extracted Data [file 12966_2019_867_MOESM2_ESM.pdf]

Supplementary File 2: Extracted Data

Table A. Shown are the extracted data points for each included article. In the case of interventions in multiple settings, only the food-retail component is described.

Authors	Study Type and Objective	Data Collected	Setting	Intervention	Intervention Outcomes	Section	QS
Adams et al. (2012)	Evaluation:<br><br>Report on process evaluation measures for the intervention	Measures of intervention fidelity, product variety, price and quality of products; experiences of intervention stakeholders	England<br><br>Small retailers:<br>87 convenience stores	*Change4Life*<br><br>Availability, promotion<br><br>9 months	Fidelity <40%; Good product quality 99%; Prices 110% of supermarket (non-substantial)	Results, discussion	MOD (0.75)
Andreyeva et al. (2010)	Evaluation:<br><br>Explore retailers' perceptions of demand for healthy foods, practices and barriers regarding stocking healthy foods, and the impact of the intervention on stores	Retailer perceptions and experiences regarding product selection, supply, pricing, demand, profitability, and attitudes, support and barriers towards carrying healthy foods	USA<br><br>Small retailers:<br>68 (baseline) and 58 (follow up) non-supermarket stores	*Supplemental Nutrition Assistance Program: Women, Infants, and Children* (government welfare programme; new guidelines)<br><br>Availability; pricing<br><br>Indefinite length, evaluation after 1 year	Retailers perceived lower demand for healthy foods at baseline and follow-up ($p<0.001$); At follow-up intervention stores reported increased demand for some healthy foods (no p value); Perceived profitability of healthy foods among intervention stores unchanged at follow-up with exception of infant formula, which increased ($p<0.05$); No significant difference in perceived profitability between intervention and control stores at baseline and follow-up<br>(non-substantial)	Results, discussion	MOD (0.65)
Ayala et al. (2015)	Design:<br><br>Describing the recruitment process and design for a prospective intervention	Baseline characteristics of participating shops, managers, and consumers	USA<br><br>Small retailers:<br>8 tiendas (small/medium Latino food stores) (control: 8 stores)	*El Valor*<br><br>Availability; promotion; POP information;<br><br>6 months	n.a.	Results	STR (0.8)
Baquero et al. (2014)	Evaluation:<br><br>Report on process evaluation measures for the intervention	Measures of intervention reach, dose delivered, dose received, and intervention fidelity	USA<br><br>Small retailers:<br>2 tiendas (small/medium Latino food stores) (control: 2 stores)	*Vida Sana Hoy y Mañana* (precursor to El Valor, see above)<br><br>Availability; promotion; POP information<br><br>16 weeks	Dose delivered was high; respondents who attended activities 60%; respondents who noticed intervention 29%; respondents who changed eating habit 60%; fidelity was high (substantial)	Results (one phrase), discussion	WEAK (0.57)
Budd et al (2015)	Design:<br><br>Describing the design for a prospective intervention	n.a.<br>*study design paper*	USA<br><br>Small retailers:<br>24 corner stores (control: 6 corner	*B'More Healthy Retail Rewards*<br><br>Availability; pricing; promotion; POP information	n.a.	Design description	STR (0.83)

			stores), 2 wholesalers	6 months			
Budd et al (2017)	Evaluation:<br><br>Evaluating the effect of the intervention on stocking, sales, and prices of an intervention	Storeowner demographic and psychosocial measures; sales, stocking, and pricing data	USA<br><br>Small retailers:<br>24 corner stores (control: 6 corner stores), 2 wholesalers	*B'More Healthy Retail Rewards*<br><br>Availability; Pricing; promotion; POP information<br><br>6 months	Increase stocking healthy foods (3.6 units, p=0.007), Increase sales healthy snacks (6.4 units/day, p=0.02), Significant decrease price healthy foods (o.47$ combined, p=0.036); Decrease expectations (3.4 units, p=0.001) for sales of promoted beverages<br>(substantial)	Discussion	STR<br>(0.88)
Fernandez et al. (2016)	Reflection:<br><br>Describe stakeholders' motivational factors for intervention adoption	Stakeholder perceptions on factors which influenced their decisions to adopt the intervention	Canada<br><br>Large retailers:<br>8 food-retailers (total: 18 cross-sectoral partners)	*Eat Well Campaign: Food Skills*<br><br>Promotion<br><br>Not specified	n.a.	Results, discussion	STR<br>(0.8)
Fernandez et al. (2017)	Reflection:<br><br>Identify barriers and facilitators for implementation experienced by stakeholders, and describe differences between them	Stakeholders' experiences in implementing the intervention	Canada<br><br>Large retailers:<br>8 food-retailers (total: 18 cross-sectoral partners)	*Eat Well Campaign: Food Skills*<br><br>Promotion<br><br>1 year	n.a.	Results, discussion	STR<br>(0.85)
Foster et al. (2014)	Evaluation:<br><br>Evaluating the effect of the intervention on healthy product sales	Sales data; measures of intervention fidelity	USA<br><br>Large retailers:<br>8 chain stores (2 chains, 4 each)	*No name*<br><br>Promotion<br><br>6 months	Significant increases in sales of skim (1509 units, p=0.0078) and 1% (3383 units, p=0.0014) milk, shelf (1690 units, p=0.0109) and cooled (19 units, p=0.0002) water , and two frozen meals (21 units, p=0.0074; 11 units, p=0.0326); Intervention fidelity overall was 64-85%<br>(substantial)	Discussion	MOD<br>(0.79)
Gardiner et al. (2013)	Reflection:<br><br>Explore barriers, facilitators, level of stakeholder participation, and perceived outcomes	Stakeholders experiences and perceptions of the intervention process and its outcomes	Australia<br><br>Small retailers:<br>6 stores (out of 13 participating)	*'improving retail access to fresh fruit and vegetables' initiative*<br>(based on Change4Life)<br><br>Availability<br><br>8 months (planned to continue up to 30)	n.a.	Results, discussion	MOD<br>(0.65)
Gittelsohn et al. (2007)[1]	Evaluation:<br><br>Report on process evaluation measures for the intervention, and impact on consumers of the intervention	Measures of consumer exposure and intervention impact on consumer diabetes knowledge, label reading knowledge, and self-efficacy, and healthy foods purchases	Marshall Islands<br><br>Small retailers:<br>unspecified number of large and small stores	*Marshall Islands Healthy Stores Program*<br><br>Unspecified<br><br>10 weeks	Consumer exposure was high for the media component, and moderate for the store component; Exposure was associated with significant changes in diabetes knowledge (p<0.05) and label reading knowledge (p<0.05); Significant increases in sales of several promoted foods (p<0.05-0.005) and	Discussion	MOD<br>(0.71)

					healthiness of cooking methods when exposed to media component (p=0.036) (substantial)		
Gittelsohn et al. (2007)[2]	Formative:<br><br>Explore the role of food stores in dietary behaviour, and other constraints for a healthy diet	Store manager/owner perceptions of the food environment and health promotion interventions	USA<br><br>Small retailers:<br>9 small food stores, 1 medium food store	*n.a.*<br><br>Formative research	n.a.	Results, discussion	MOD (0.75)
Gittelsohn et al. (2010)	Evaluation:<br><br>Report on process evaluation measures for the intervention	Measures of intervention fidelity, reach and dose	USA<br><br>Mixed retailers:<br>2 supermarkets, 7 corner stores	*Baltimore Healthy Stores*<br><br>Availability; pricing; promotion; POP information<br><br>10 months	High overall availability of products (mean 86%); Varying fidelity of printed material placement (9-95% across phases); low fidelity, dose and reach for coupons (13-25% handed out and reimbursed); high taste test fidelity (179% of target) with high dose delivered (140% of target per participant) and achieving target number of interactions (2942) (substantial)	Results, discussion	WEAK (0.58)
Gittelsohn et al. (2012)	Reflection:<br><br>Explore small store-retailers' perceptions of the intervention	Store manager/owner experiences with the implementation of the intervention	USA<br><br>Small retailers:<br>52 small urban food stores	*Supplemental Nutrition Assistance Program: Women, Infants, and Children* (government welfare programme; new guidelines)<br><br>Availability; pricing<br><br>Indefinite length	n.a.	Results, discussion	MOD (0.75)
Gittelsohn et al. (2013)	Evaluation:<br><br>Report on process evaluation measures for the intervention	Measures of intervention fidelity, reach and dose	USA<br><br>Small retailers:<br>21 stores/ carryouts	*Baltimore Healthy Eating Zones*<br><br>Availability; promotion; POP information<br><br>10 months	Overall moderate fidelity of stocking foods (mean 74%, range 0-100%); low/moderate fidelity for shelf materials (mean 30%); overall moderate fidelity for posters (mean 81%); Moderate/high dose delivered for interactive session/giveaways (2.8 components per participant), and reach of mean 15 interactions per visit (substantial)	Discussion	STR (0.86)
Gittelsohn et al. (2014)[1]	Design:<br><br>Describing the design for a prospective intervention	n.a.<br>*study design paper*	USA<br><br>Small retailers:<br>2 local wholesalers, 1 national wholesaler, 60+ corner stores (2+ per study zone)	*B'More Healthy Communities for Kids*<br><br>Availability; promotion; POP information<br><br>Unspecified length	n.a.	Design description	MOD (0.67)
Gittelsohn et al.	Reflection:	Interventionists' experiences regarding the challenges	USA	*1: Baltimore Healthy Stores*<br>Availability; pricing; promotion; POP	n.a.	Results, discussion	MOD (0.65)

(2014)[2]	Reflect on challenges encountered and lessons learned by several interventions in small stores	encountered by interventions in small stores, and how these challenges were addressed	Mixed retailers:<br>5 cases<br>(1: 4 supermarkets and 28 stores)<br>(2: 18 corner stores)<br>(3: 39 stores)<br>(4: 2 stores)<br>(5: 640 stores)	information<br>1 year<br><br>*2: Baltimore Healthy Eating Zones*<br>Availability; promotion; POP information<br>2 years<br><br>*3: Minneapolis Healthy Corner Store program*<br>Availability; promotion<br>2 years<br><br>*4: Vida Sana Hoy y Mañana*<br>Promotion; POP information<br>Not specified<br><br>*5: Food Trust's Healthy Corner Store Initiative*<br>Availability; promotion; POP information<br>2 years			
Gudzune et al. (2015)	Evaluation/reflection:<br><br>Report on process evaluation measures for the intervention, its outcomes, and evaluating stakeholder perceptions on intervention process and execution	Variety, quantity and costs of delivered products; price, remaining products and spoiled products in shop; retailers experiences with the intervention	USA<br><br>Small retailers:<br>2 farm-store pairs	*No name*<br><br>Availability<br><br>9 weeks	In store 1 the number of products carried increased with 11.3 (p=0.01), and 86% of delivered products were sold; In store 2 number of products carried did not increase significantly (2.2, p=0.44) and 63% of delivered products were sold<br>(substantial)	Results, discussion	WEAK<br>(0.59)
Jetter et al. (2010)	Evaluation:<br><br>Explore the whether offering fresh produce as a retailer is commercially viable if the barrier of start-up costs is covered	Sales data; retailers' perspectives on the intervention	USA<br><br>Small retailers:<br>1 convenience store	*No name*<br><br>Pricing ; unspecified "minor improvements"<br><br>31 weeks	The sales of fresh produce was positively associated with number of weeks into the intervention, number of items available, pounds of fresh produce available, and the distribution of flyers, it was negatively associated with some other than the owner being responsible for restocking the fresh produce (all $p \leq 0.05$)<br>(substantial)	Results, discussion	STR<br>(0.82)
Johnston et al. (2014)	Reflection:<br><br>Describe the implementation of the intervention, and lessons learned regarding this process	Experiences from the involved interventionists	USA<br><br>Large retailers:<br>11 supermarket chain stores	*Broome County Sodium Reduction in Communities Project*<br><br>Promotion<br><br>Not specified	n.a.	Discussion	WEAK<br>(0.5)
Karpyn et al. (2018)	Formation:<br><br>Explore potential barriers and	Store inventory data; perceptions of retailer on implementing the intervention	USA<br><br>Small retailers:	*Supplemental Nutrition Assistance Program* (government welfare programme; new guidelines)	n.a.	Results	STR<br>(0.9)

	facilitators for the implementation of the intervention, and compare current stocking practices with the intervention requirements		57 small stores	Availability<br><br>Indefinite length			
Kim et al. (2017)	Formation:<br><br>Understand what barriers or facilitators retailers perceive for stocking and promoting healthy foods, and commonalities and differences in reasoning regarding promoting healthy food	Retailers perceptions of barriers, opportunities, and strategies for the stocking of healthy foods	USA<br><br>Small retailers:<br>15 small stores	*n.a.*<br><br>Formative research	n.a.	Results, discussion	STR (0.8)
Lee et al. (2015)	Evaluation:<br><br>Report on process evaluation measures for the intervention, explore barriers and facilitators for implementation, and asses sustainability	Measures of intervention fidelity, reach, dose; impact on employee behavioural intention and self-efficacy	USA<br><br>Large retailers:<br>1 supermarket	*Eat Right-Live Well!*<br><br>Availability; promotion; POP information<br><br>8 months	Fidelity was high for item stocking (m88%, range 66-93%) , and moderate for labelling (mean 71%, range 49-81%) and visible advertisements (mean 75%, range 26-97%); for taste test sessions reach was mean 30 (range 23-35) and dose was mean 10.5 (range 2-21) items given away per session; Community event reached mean 62 participants per month; Employee measures did not change significantly (substantial)	Results	MOD (0.67)
Lent et al. (2014)	Evaluation:<br><br>Evaluating the impact of the intervention on participant health status and purchasing behaviour	Participant height and weight; energy content of purchases in stores by participants	USA<br><br>Small retailers:<br>10 schools and 24 surrounding corner stores (5 school and 12 stores control)	*No name*<br><br>Availability; promotion<br><br>2 years	No significant changes in energy content per purchase, BMIz scores, or obesity prevalence (non-substantial)	Discussion	STR (0.92)
Mah et al. (2017)	Formation:<br><br>Examine strategies used and barriers/facilitators perceived by food environment actors regarding health promotion	Food-environment actors' experiences with carrying out their everyday work, and opportunities and constraints to promoting health in their environment	Canada<br><br>Small retailers:<br>25 entrepreneurial actors from two studies on facilitating a healthy food environment (9 small business owners, including retailers)	*Food Retail Environments Shaping Health*<br><br>"healthy corner store" conversion (no details given)<br><br>*Missing Middle*<br><br>Search for policy levers for healthy food environment	n.a.	Results, discussion	MOD (0.75)
Mayer et al. (2016)	Formation:<br><br>Gather retailer perceptions on community health issues, their	Retailer perceptions on community views of chronic diseases and diet, and the role of stores in the community;	USA<br><br>Small retailers:<br>23 stores	*Healthy Corner Store Initiative Formative*<br><br>Availability; promotion	n.a.	Results, conclusion	STR (0.85)

	own role in the community, and the challenges of operating as a retailer	retailers' challenges experienced in their work		Not specified			
Mead et al. (2010)	Formation:<br><br>Explore the influence of food environment, perceptions of health and food, history, culture, and perceived barriers to eating healthy, on dietary behaviour	Interviews with community members and retailers regarding their dietary behaviour, influential factors, and barriers to eating healthy	Canada (Inuit communities)<br><br>Small retailers: 2 local stores	*n.a.*<br><br>Formative research	n.a.	Results	STR (0.8)
Mead et al. (2013)	Evaluation:<br><br>Evaluating the impact of the intervention on participant psychosocial determinants of diet, food-related behaviour, and weight status	Demographic characteristics; Measures of food knowledge, and self-efficacy and intentions to eat healthy	Canada (Inuit communities)<br><br>Small retailers: 12 stores	*Healthy Foods North*<br><br>Availability; promotion; POP information;<br><br>1 year	Respondents living in the intervention communities showed improved food-related self-efficacy (p=0.003), and intentions to eat healthy (p=0.001) compared to those in control communities<br>(substantial)	Discussion	MOD (0.79)
Närhinen et al (2000)	Evaluation:<br><br>Evaluating the feasibility of using sales data to measure intervention impact	Sales data of targeted products	Finland<br><br>Large retailers: 1 supermarket	*No name*<br><br>Promotion; POP information<br><br>12 weeks	No significant changes were observed in the sales of intervention targeted products<br>(non-substantial)	Discussion	MOD (0.64)
Novotny et al. (2011)	Evaluation:<br><br>Report on process evaluation measures for the intervention, and stakeholders' experiences with the intervention	Intervention reach, dose, and fidelity; perspectives food producers and distributors	USA<br><br>Small retailers: 5 stores	*Healthy Foods Hawai'i*<br><br>Promotion; POP information<br><br>34-42 weeks	Educational materials achieved moderate to high fidelity with one low point(range 33-100%), product stocking achieved moderate to high fidelity (range 75-100%); cooking demonstrations achieved a reach of 1154 participants in total, and a dose delivered of 713-869 samples per phase<br>(substantial)	Results	STR (0.86)
Paek et al (2014)	Evaluation:<br><br>Evaluating the impact of the intervention on the availability, price, and quality of healthy foods, and consumer awareness and shopping behaviour	In-store healthy food accessibility, and consumers' consumption and purchase patterns, awareness of store changes, and intervention awareness	USA<br><br>Small retailers: 4 stores	*FIT Store Program*<br><br>Availability; promotion; POP information<br><br>6 months	Product availability increased in 3/4 stores, product variety increased or stayed constant, and affordability changes varied; Respondent awareness of the project increased (p=0.05); bean and nut consumption increased (p<0.01); 64.9-80.0% of respondents noted changes in the stores; 38.0-46.3% of respondents reported dietary changes<br>(substantial)	Discussion	MOD (0.77)
Pitts et al. (2013)	Formation:<br><br>Examine the feasibility of an	Retailers perceptions of their customers' acceptance of government welfare program	USA<br><br>Small retailers:	*n.a.*<br><br>Formative research	n.a.	Results, discussion	MOD (0.7)

	intervention design, and collect baseline data for the intervention evaluation	benefits, store products and inventory, availability of healthy products, and the community role of the store and interest in intervention participation;<br>Customer shopping and eating habits, product needs,	11 stores				
Pothukuchi et al. (2016)	Reflection:<br><br>Discuss the experiences of and lessons learned from an intervention pilot	Community stakeholder perspectives and interventionist experiences regarding the implementation of an intervention	USA<br><br>Small retailers:<br>26 corner stores	*Detroit FRESH*<br><br>Availability<br><br>5 years	n.a.	Results, Discussion	MOD (0.6)
Rosecrans et al. (2008)	Evaluation:<br><br>Report on process evaluation measures for the intervention, its feasibility, and opportunities for improvement	Evaluative measures of intervention fidelity, dose delivered, dose received, reach, and contextual influences on the intervention process, and the experiences of intervention deliverers	USA (native communities)<br><br>Mixed retailers:<br>3 small stores, 4 medium stores, 1 supermarket	*Zhiiwaapenewin Akino'maagewin: Teaching to Prevent Diabetes*<br><br>Availability; promotion; POP information<br><br>40 weeks	The store components achieved 70% fidelity for the availability of promoted products, 60% fidelity for shelf label placement, 71% fidelity for cooking demonstrations with a total reach of 572 people and a dose of m20 hand-outs per demonstration<br>(substantial)	Results, discussion	MOD (0.67)
Rushakoff et al. (2017)	Evaluation:<br><br>Evaluating the impact of the intervention on the availability of healthy foods, and consumer eating and purchasing habits	Nutrition environment descriptive measures; store inventory logs; retailer experiences; community purchasing and dietary behaviours	USA<br><br>Small retailers:<br>10 rural stores	*Healthy2Go*<br><br>Availability; promotion; POP information<br><br>18 months	Program participating stores achieved a 40% increase in fresh produce stocking and 20% increase in product variety; self-reported healthy food consumption increased ($p<0.05$)<br>(substantial)	Discussion	MOD (0.64)
Schwendler er al (2017)	Evaluation:<br><br>Report on process evaluation measures for the intervention	Store environment assessment; evaluative store-visit assessment; measures of intervention fidelity, reach and dose	USA<br><br>Small retailers:<br>2 local wholesalers, 1 national wholesaler<br>29 corner stores (control: 24 corner stores)	*B'More Healthy Communities for Kids*<br><br>Availability; promotion; POP information<br><br>6 months	At the wholesale level intervention reach was high (range 100-150% of target) and fidelity improved from low to high (range 74-124%); At the store level reach was moderate (range 78-81%), dose delivery increased from moderate to high (85-101%), fidelity increased from moderate to high (range 85-112%)<br>(substantial)	Discussion	STR (0.85)
Song et al. (2009)	Evaluation:<br><br>Report on intervention acceptability to retailers, necessary operational changes, and commercial sustainability	Intervention impact on retailer psychosocial variables, stocking and sales, and promoted foods	USA<br><br>Mixed retailers:<br>2 supermarkets, 7 (Korean) corner stores<br>(control: 2 supermarkets, 6 (Korean) corner stores)	*Baltimore Healthy Stores*<br><br>Availability; pricing; promotion; POP information<br><br>9 months	Intervention-store retailers' expectations for the sales of healthy crackers were higher then those of comparison stores ($p=0.04$), as was their self-efficacy for stocking low sugar cereals ($p=0.01$); Stocking of promoted products was higher for intervention stores at post-phase ($p=0.004$) and post-intervention	Discussion	MOD (0.64)

					(p=0.009), as were sales of promoted products in post-phase (p=0.05) and post-intervention (p=0.003) (substantial)		
Song et al. (2011)	Reflection:<br><br>Exploring motivational factors, implementation success, and factors of influence on retailers' support for the intervention	Open interviews with retailers; field notes and evaluations of the execution of the intervention by retailers	USA<br><br>Small retailers:<br>7 (Korean) corner stores (control: 6 (Korean) corner stores)	*Baltimore Healthy Stores*<br><br>Availability; pricing; promotion; POP information<br><br>10 months	n.a.	Results, discussion	MOD (0.7)
Steenhuis et al. (2001)	Formation:<br><br>Identify factors of influence on the adoption, and implementation of interventions	Retailers perceptions regarding intervention characteristics and requirements, and organisational characteristics	Netherlands<br><br>Large retailers:<br>8 supermarkets	*n.a.*<br><br>*Formative research*	n.a.	Results	MOD (0.6)
Steenhuis et al. (2004)	Evaluation/reflection:<br><br>Evaluate the implementation process and identify possible reasons for the limited impact of the intervention	Retailers' perceptions of the intervention components, their implementation, organisation, and impact on customers	Netherlands<br><br>Large retailers:<br>9 supermarkets	*No name*<br><br>Promotion; POP information<br><br>6 months	Allegedly low impact, not specified further<br>(non-substantial)	Results	MOD (0.65)
Young et al. (2017)	Reflection:<br><br>Discuss successes and challenges of the intervention process.	Measures of store offer in local products; retailer perceptions of organizational capacity; interviews with retailers	USA<br><br>Small retailers:<br>5 corner stores	*The Lindsay Heights Healthy Corner Store Initiative*<br><br>Availability; promotion<br><br>2 years	n.a.	Results, discussion	WEAK (0.3)

Intervention typology legend

Availability	Interventions which change the availability, variety and convenience of healthy products in the store, e.g. through stocking more products, or adjusting their placement on the shelves.
Pricing	Interventions which change the price of products, e.g. increases/decreases, discounts, or coupons.
Promotion	Interventions related to the promotion of certain products through media advertisements, commercials, tasting demonstrations, or posters
Point-of-purchase information (POP)	Interventions related to the provision of information at the point-of-purchase (e.g. shelf tags,) meant to identify healthy choices
